# Late-onset hypersensitivity after a lesion in the ventral posterolateral nucleus of the thalamus: A macaque model of central post-stroke pain

**DOI:** 10.1038/s41598-017-10679-2

**Published:** 2017-09-04

**Authors:** Kazuaki Nagasaka, Ichiro Takashima, Keiji Matsuda, Noriyuki Higo

**Affiliations:** 1 Human Informatics Research Institute, National Institute of Advanced Industrial Science and Technology (AIST), Tsukuba, Ibaraki 305-8568 Japan; 2 Graduate School of Comprehensive Human Sciences, University of Tsukuba, Tsukuba, Ibaraki 305-8577 Japan

## Abstract

Central post-stroke pain (CPSP) can occur as a result of a cerebrovascular accident in the ventral posterolateral nucleus (VPL) of the thalamus. Developing therapeutic interventions for CPSP is difficult because its pathophysiology is unclear. Here we developed and characterized a macaque model of CPSP. The location of the VPL was determined by magnetic resonance imaging (MRI) and extracellular recording of neuronal activity during tactile stimulation, after which a hemorrhagic lesion was induced by injecting collagenase type IV. Histological analysis revealed that most of the lesion was localized within the VPL. Several weeks after the injection, the macaques displayed behavioral changes that were interpreted as reflecting the development of both mechanical allodynia and thermal hyperalgesia. Immunohistochemistry revealed that microglial and astrocytic activation in the perilesional areas lasted at least 3 months after injection. The present model reproduced the symptoms of patients suffering from CPSP, in which both mechanical allodynia and thermal hyperalgesia often develop several weeks after cerebrovascular accident. Further, the long-lasting glial activation revealed here may be characteristic of primate brains following injury. The present model will be useful not only for examining the neurological changes underlying CPSP, but also for testing therapeutic interventions for CPSP.

## Introduction

Central post-stroke pain (CPSP) is a chronic neuropathic pain that occurs as a result of a stroke lesion in the somatosensory pathway that includes the posterolateral region of the thalamus^1–4^. CPSP is characterized by not only spontaneous pain but also evoked pain in which normally innocuous stimuli are perceived as painful, *i.e*., allodynia, or normally painful stimuli are perceived as even more painful, *i.e*., hyperalgesia. CPSP occurs in up to 10% of stroke patients; it decreases the quality of life and frequently interferes with rehabilitation of the affected patients^2^. However, developing therapeutic interventions for CPSP is difficult at present because its pathophysiology remains to be revealed.

In recent years, rodent models of CPSP have been developed to address this problem. In these models, a lesion of the thalamus including the VPL was made by inducing a focal stroke. Using these models, researchers have shown that a lesion around the VPL is associated with allodynia- and hyperalgesia-like behavior and have suggested that some drugs can ameliorate the symptoms.

In addition to these rodent models, a primate model of CPSP, using rhesus macaques, might also contribute to overcoming CPSP because it is more compatible with humans in regard to the structures and functions of brain regions suggested to be involved in pain in humans. For example, the primary somatosensory cortex, which is associated with sensory aspects of pain including localization of painful stimuli and discrimination of its intensity^5^, is well developed in rhesus macaques and clearly differentiated into four subdivisions, *i.e*., areas 3a, 3b, 1, and 2—a subdivision not observed in rodents^6^. Moreover, rhesus macaques and humans share homologous anatomical and physiological features of the prefrontal cortex^7, 8^, which is involved in the cognitive-evaluative aspects of pain^9, 10^. In contrast, several studies have suggested that there are no obvious homologues of prefrontal cortex in rodents^11, 12^. Finally, a recent anatomical brain imaging study in human patients suggested the involvement of the anterior pulvinar nucleus, a highly developed brain structure in primates including macaques, in CPSP^13^. The homology of pain-related areas between rhesus macaque and human suggests that the macaque CPSP model could be advantageous in translating findings into clinically useful treatments for CPSP.

The present study aimed to develop a macaque model of CPSP by inducing a hemorrhage in the VPL. Although CPSP can develop after both ischemic and hemorrhagic vascular lesions^14–16^, we focused on hemorrhagic stroke in the present study because most rodent CPSP models are created by inducing hemorrhage^17–22^; therefore, we can investigate how symptoms after thalamic damage differ between rodents and primates. In addition, we performed histological analyses to investigate both microglial and astrocytic activation, which is associated with the development of abnormal pain after peripheral nerve and spinal cord injury^23–27^.

## Results

### Stroke and lesion sizes

**Figure 1:**
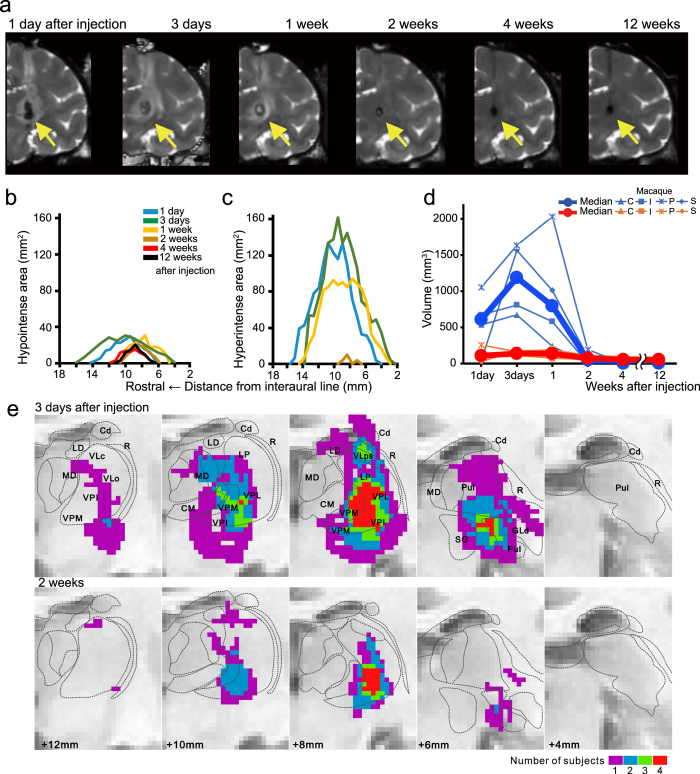
Time course of stroke after injection. (**a**) Coronal T2-weighted MR images of Macaque I showing time course of stroke after the injection. The area that contained a hematoma and edema was seen as the hypointense stroke core (arrows) and the surrounding hyperintense rim. The coronal images were obtained through the rostro-caudal level at which the VPL is located. (**b**,**c**) Temporal changes of the hypointense (**b**) and hyperintense areas (**c**) in successive coronal images of Macaque I are shown as a function of rostral distance from the interaural line. In this macaque, 4 to 17 mm rostral to the interaural line corresponds to the level at which the thalamus is located. (**d**) Temporal volume changes of the hypointense (red) and hyperintense areas (blue) are shown for all four collagenase-injected rhesus macaques. In all animals, the areas of both hypointense and hyperintense signal intensity decreased at 2 weeks after the collagenase injection, when the hyperintense area almost disappeared. Thereafter, the residual hypointense area was almost stable until 12 weeks after injection. (**e**) Using a normalized MRI brain template and atlas-based borders indicated that the stroke area at 3 days after collagenase injection included the ventral posterolateral nucleus (VPL) and several additional thalamic nuclei. However, at 2 weeks after injection and thereafter, the stroke was mainly localized within VPL. The colors indicate the number of macaques with hypointense signals in the indicated voxels. Cd, caudate nucleus; CM, central medial nucleus; GLd, dorsal lateral geniculate nucleus; LD, lateral dorsal nucleus; LP, lateral posterior nucleus; MD, dorsomedial nucleus; Pul, pulvinar nucleus; R, reticular nucleus; SG, suprageniculate nucleus; VLo, oral part of the ventrolateral nucleus; VLc, caudal part of the ventrolateral nucleus; VLps, ventrolateral nucleus, pars postrema; VPM, ventral posteromedial nucleus; VPI, ventral posteroinferior nucleus.

**Figure 2:**
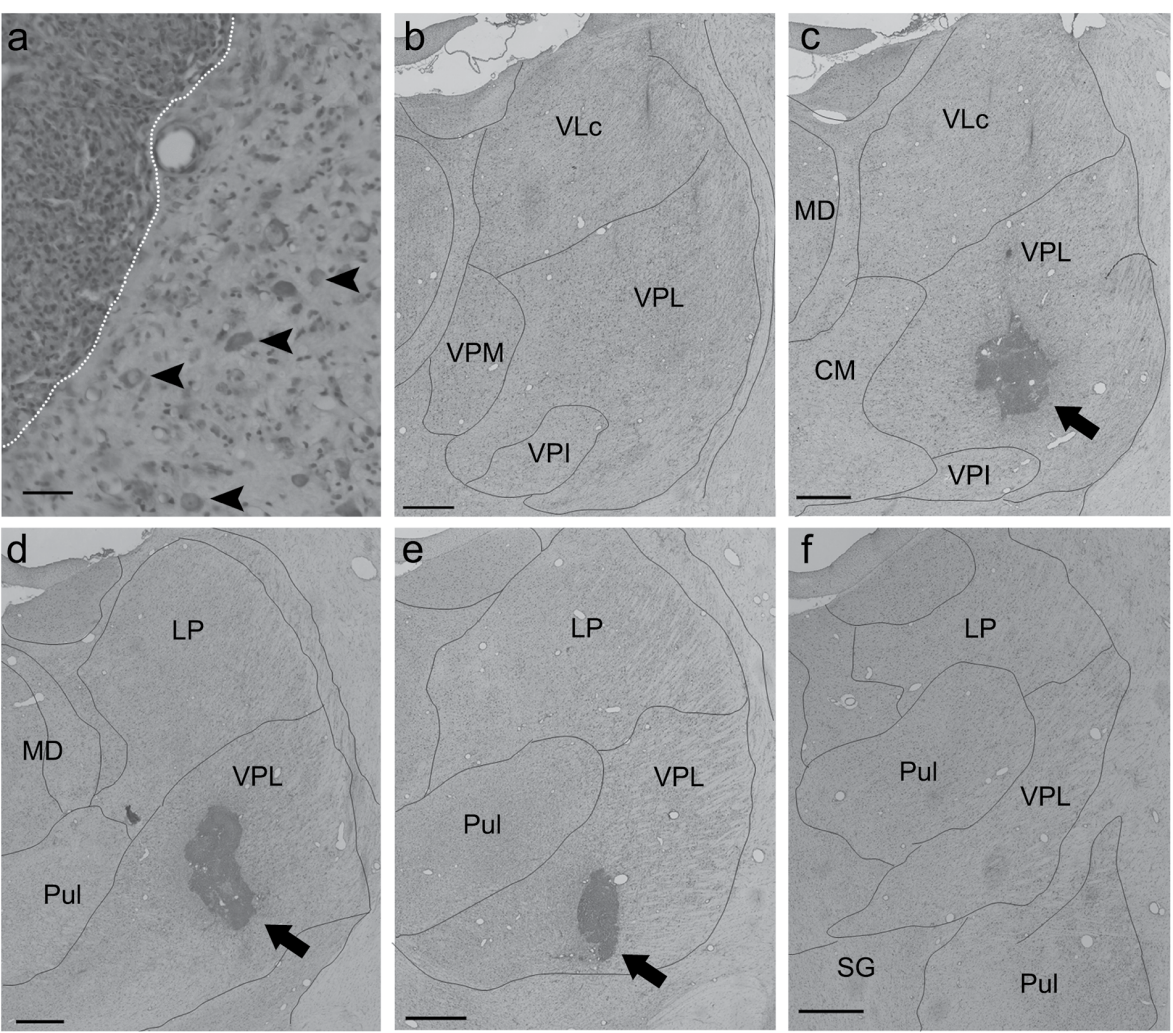
Confirmation of lesional area. (**a**) A Nissl-stained coronal section showing both the lesional and perilesional areas in the VPL. The lesional area was defined by a dense concentration of small cells (5–10 μm in diameter), which presumably includes both glial cells and blood cells. The boundary between the lesional and perilesional areas is indicated by the white dotted line (left, lesional area; right, perilesional area). Large cells (20–40 μm in diameter, arrowheads) presumed to be neurons were observed in the perilesional area. Scale bar: 50 μm. (**b**–**f**) Serial Nissl-stained coronal sections of Macaque I, spaced by approximately 600 μm, are arranged from rostral (**b**) to caudal (**f**). Note that the lesion was located within the VPL. Scale bar: mm. CM, central medial nucleus; LP, lateral posterior nucleus; MD, dorsomedial nucleus; Pul, pulvinar nucleus; SG, suprageniculate nucleus; VLc, caudal part of the ventrolateral nucleus; VPM, ventral posteromedial nucleus; VPL, ventral posterolateral nucleus; VPI, ventral posteroinferior nucleus.

A T2-weighted MR image of a rhesus macaque (Macaque I) 1 day after collagenase injection shows that the stroke area, which was seen as a hypointense core and a surrounding hyperintense rim, was mainly localized around the thalamus (arrow in Fig. 1a). The areas of both hypointense and hyperintense signal intensity expanded from day 1 to day 3 after the injection; this may result from expansion of the hematoma and edema (Fig. 1b,c). These areas then decreased rapidly over the course of 2 weeks, when the hyperintense area almost disappeared. Thereafter, the residual hypointense area was almost stable until the end of the experiment 3 months after injection (Fig. 1b). T2-weighted MR images of the other three macaques (Macaques C, P and S) also indicated a stroke mainly localized around the thalamus, and the volume dynamics were similar to that in Macaque I (Fig. 1d). A normalized MRI brain template with atlas-based borders indicated that the stroke area at 3 days after collagenase injection, when the stroke was largest, included several other thalamic nuclei in addition to VPL (Fig. 1e). By contrast, the stroke area was mainly localized within VPL ≥ 2 weeks after injection. The collagenase-induced lesion was also histologically estimated by using Nissl-stained coronal sections (Fig. 2). We defined the lesioned area as the area of a dense concentration of small cells (5–10 μm in diameter) (Fig. 2a), which presumably include both glial cells and blood cells^28^. The serial coronal sections of Macaque I indicated that the lesion was located within the VPL of the thalamus (arrows in Fig. 2c–e). The histological lesion was also localized within the VPL of Macaque C. In Macaque S, however, lesions were observed not only in the VPL but also in adjacent thalamic nuclei, *i.e*., the caudal part of the ventral lateral nucleus (32% of the total lesion volume) and the reticular nucleus (4%), as well as in other brain areas including the caudate nucleus (17%, Table 1).

**Table 1:** Collagenase-injected rhesus macaques used in the present study.

	Weight, kg	Injected hemisphere	Dosage of collagenase, μl	Lesion volume, mm^3^	Post-injection survival period, days
Macaque C	8.5	Right	4	2.1	104
Macaque I	8.0	Left	8	3.9	92
Macaque P	8.9	Left	8	−*	—
Macaque S	8.6	Right	16	7.3 (1.5)**	113

*Data is not available because the macaque is not killed for this study. **The lesion volume outside the thalamus is shown in parentheses.

### Behavioral changes after collagenase injection

**Figure 3:**
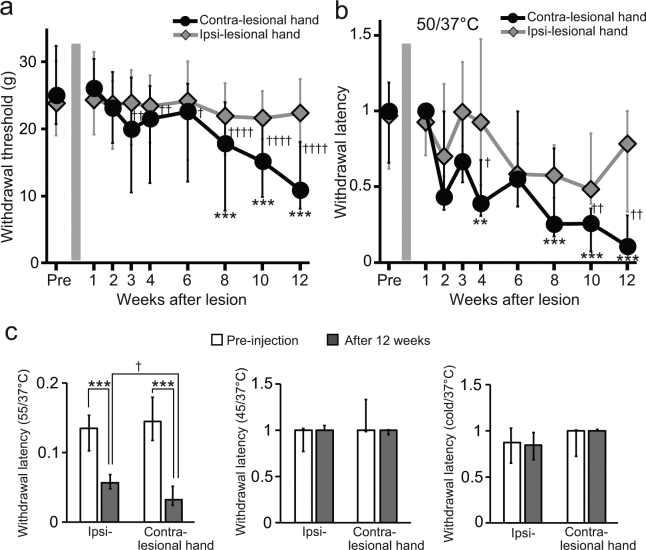
Behavioral changes after collagenase injection. (**a**,**b**) Weekly changes in the withdrawal response to mechanical and thermal stimulation. The withdrawal thresholds, shown as the pressure exerted (in grams, (**a**), and the latencies, represented as the ratio of the withdrawal latency for thermal stimulation (50 °C) to that for control stimulation (37 °C), (**b**), are shown for both hands, one contralateral (contra-lesional hand) and the other ipsilateral (ipsi-lesional hand) to the injected hemisphere. Medians and interquartile ranges for the three collagenase-injected macaques are shown. Several weeks following the injection, both the withdrawal threshold for mechanical stimulation and withdrawal latency for thermal stimulation on the contralateral hand significantly decreased relative to those before the injection (Supplementary Video S1 and S2), but not observed on the ipsilateral hand. The reductions lasted until the end of the behavioral experiment 3 months after the injection. **P < 0.01 and ****P < 0.0001, compared with pre-injection (Kruskal–Wallis one-way ANOVA followed by Dunn’s *post hoc* test). ^†^ *P* < 0.05, ^††^ *P* < 0.01, and ^††††^ *P* < 0.0001, compared with ipsi-lesional hand (Wilcoxon signed-rank test). (**c**) Medians and interquartile ranges for the withdrawal latency for thermal stimulations other than 50 °C before and at 12 weeks after injection. The withdrawal latencies for 55 °C on the both contra- and ipsi-lesional hands significantly decreased relative to those before injection (****P < 0.0001, Mann-Whitney U test), although the latency on the ipsi-lesional hand was longer than that on the contra-lesional hand at 12 weeks after injection (^†^ *P* < 0.05, Wilcoxon signed-rank test).

To determine whether a VPL lesion alters pain perception, we evaluated withdrawal responses to both mechanical and thermal stimuli applied to the surface of the hands of the macaques before and after collagenase injection. Before the injection, the withdrawal responses to both mechanical and thermal stimuli were almost identical for both hands (Fig. 3a,b). Moreover, the median withdrawal latencies for thermal stimulation at 50 °C did not significantly differ from those for control stimulation (37 °C) (*P* = 0.8646, Mann–Whitney U-test, for the hand contralateral to the hemisphere to be subjected to collagenase injection), and the ratios of the withdrawal latency for thermal stimulation to that for control stimulation before injection were close to 1 for both hands (Fig. 3b), indicating that the macaques consistently responded to each stimulus.

During the first 2–3 days after collagenase injection, all four macaques showed mild paralysis of the hind-limb contralateral to the injected hemisphere. This may be due to the fact that the posterior internal capsule where pyramidal tracts control hind-limb movements was temporarily affected by stroke because it is located adjacent to the VPL^29^. There was no apparent impairment of movements for other body parts, such as the forelimb and face, and the macaques showed no difficulty in grasping a piece of food or in eating and drinking. Moreover, the withdrawal responses to both mechanical and thermal stimuli during the first week after injection were almost identical to those before injection (Fig. 3a,b), indicating that the macaques’ hand movements during the withdrawal tests were not affected by the stroke. The lack of motor paralysis in the forelimb indicates that stroke spared the portion of the posterior internal capsule where pyramidal tracts control forelimb movements; this site is located several millimeters rostral to VPL^29^.

A Kruskal–Wallis test revealed significant changes in both the withdrawal threshold for mechanical stimulation and the ratio of the withdrawal latency for thermal stimulation to that for control stimulations on the hand contralateral to the injected hemisphere (*i.e*., contra-lesional hand) across weeks after the injection (Fig. 3a,b, *P* < 0.0001, respectively). Dunn’s *post hoc* analyses demonstrated a statistically significant reduction in both the withdrawal threshold for mechanical stimulation (Fig. 3a, *P* < 0.0001) at 8 weeks and the ratio of the withdrawal latency for thermal stimulation to that for control stimulation (Fig. 3b, *P* = 0.0051) at 4 weeks after injection compared with those before injection, respectively. The behavioral changes were interpreted as indicating the development of both mechanical allodynia (Supplementary Video S1) and thermal hyperalgesia (Supplementary Video S2). We cannot conclude definitively that thermal hyperalgesia develops earlier than mechanical allodynia because the withdrawal latency for the contra-lesional hand in response to thermal stimulation became significantly lower than that for the ipsi-lesional hand at a later time than the withdrawal thresholds in response to mechanical stimulation became significantly different for the 2 hands (Fig. 3a,b). The reductions lasted until the end of the behavioral experiments, 3 months after the injection. There was no correlation between the degree of the reduction and the dosage of collagenase, *e.g*., the ratios of the withdrawal threshold at 12 weeks after the injection to that before injection were 0.68, 0.27, 0.51, and 0.55 in Macaques C (4-μl), I (8-μl), P (8-μl), and S (16-μl), respectively. We did not observe significant change for mechanical withdrawal responses on the hand ipsilateral to the injected hemisphere, *i.e*., the ipsi-lesional hand (Fig. 3a). Although the median ratio of the withdrawal latency on the ipsi-lesional hand for thermal stimulation at 50 °C to that for control stimulation showed a tendency to decrease, the decrease was not statistically significant (Fig. 3b). Even though no motor paralysis was detected after collagenase injection, all four macaques rarely used the contra-lesional hand to retrieve food in their cages; this behavior suggests that mechanical allodynia developed in the contra-lesional hand. We also evaluated changes in withdrawal latency for thermal stimulation at temperatures other than 50 °C (Fig. 3c). No significant change was observed for withdrawal latency on either the contra- or ipsi-lesional hands for thermal stimulation at 45 °C or cold stimuli (10–5 °C) at 3 months after the injection. The withdrawal latency for thermal stimulation at 55 °C compared with that for control stimulation before injection was less than 1 for both hands, indicating that the intact monkeys sensed thermal stimulation at 55 °C as a nociceptive stimulus. We measured withdrawal latency to thermal stimulation at 55 °C to confirm that the macaques remained able to withdraw in response to nociceptive stimuli after collagenase injection. The withdrawal latency to stimulation at 55 °C did not increase after collagenase injection, suggesting a lack of motor impairment. Rather, it significantly decreased for both hands at 3 months after the injection compared with the value before injection (*P* < 0.0001, Mann–Whitney U-test), although the latency on the ipsi-lesional hand was longer than that on the contra-lesional hand at 3 months after injection (*P* = 0.0464, Wilcoxon signed-rank test). These results suggest that mild hypersensitivity to heat stimuli occurred in the ipsi-lesional hand.

### Glial activation

**Figure 4:**
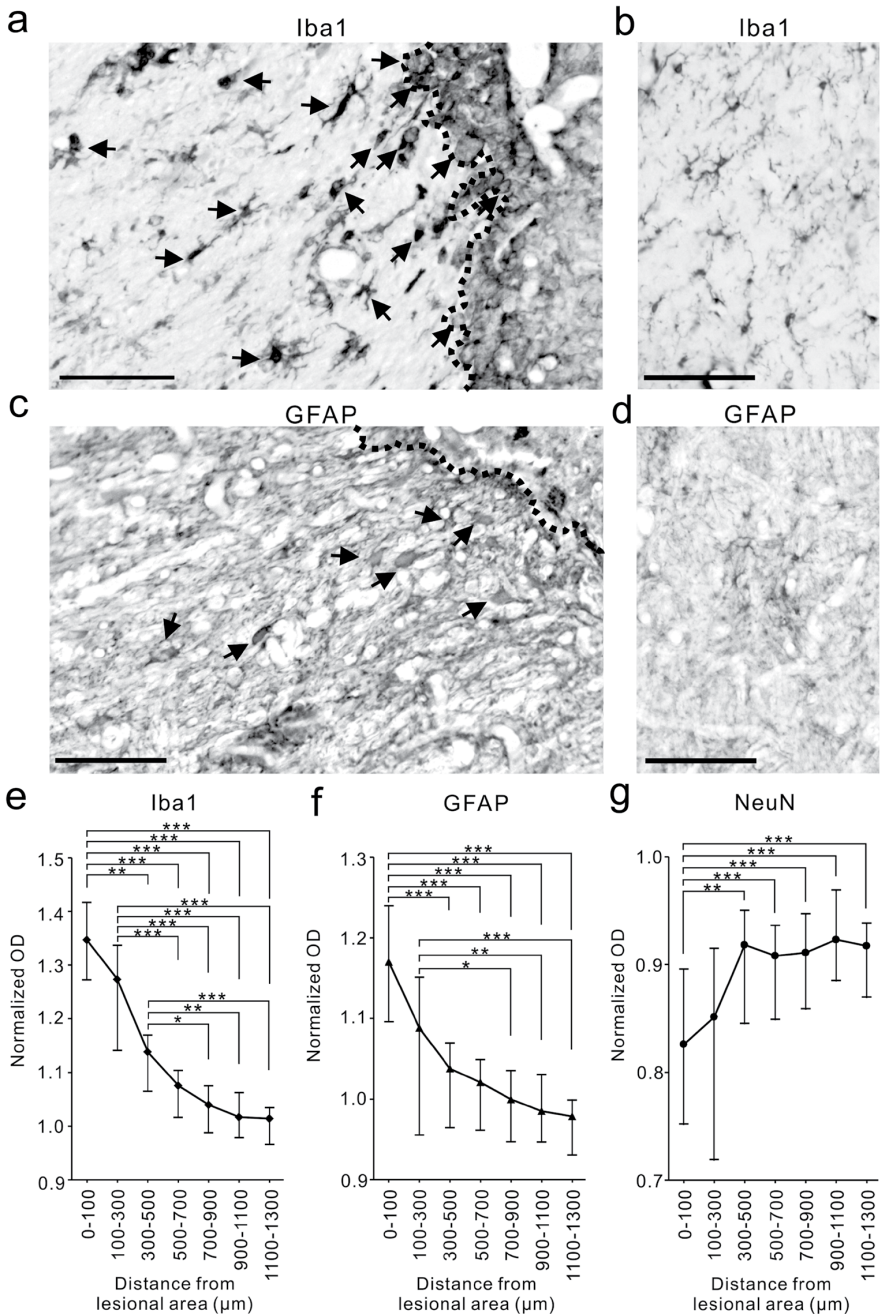
Glial activation in the perilesional area at 3 months after collagenase injection. (**a**–**d**) The coronal sections showing localization of ionized calcium binding adaptor molecule 1 (Iba-1) and glial fibrillary acidic protein (GFAP) in the VPL of both the collagenase-injected (**a**,**c**) and contralateral hemispheres (**b**,**d**). (**a**) There existed Iba-1-positive cells with characteristic shapes of activated microglia, *i.e*., enlarged cells with thickening of proximal processes and reduced ramification of distal branches (arrows), in the perilesional area (the left side of the dotted line). (**b**) No activated microglia was detected in the VPL of the contralateral hemisphere. (**c**,**d**) GFAP-positive cells with characteristic shapes of activated astrocytes, *i.e*., enlarged cells with thickening of proximal processes were observed in the perilesional area (allows in **c**), but not observed in the contralateral hemisphere (**d**). Scale bar: 100 μm. (**e**–**g**) Immunoreactivities of Iba-1 (**e**), GFAP (**f**) and NeuN immunoreactivity (**g**) in the perilesional area are shown as a function of distance from the border of the lesional area. The optical density (OD) of immunoreactivity was normalized to the values obtained from the VPL of the contra-lesional hemisphere and those in the corresponding VPL of normal intact macaques. *P < 0.05, **P < 0.01 and ***P < 0.001 (Kruskal–Wallis one-way ANOVA followed by Dunn’s *post hoc* test).

**Figure 5:**
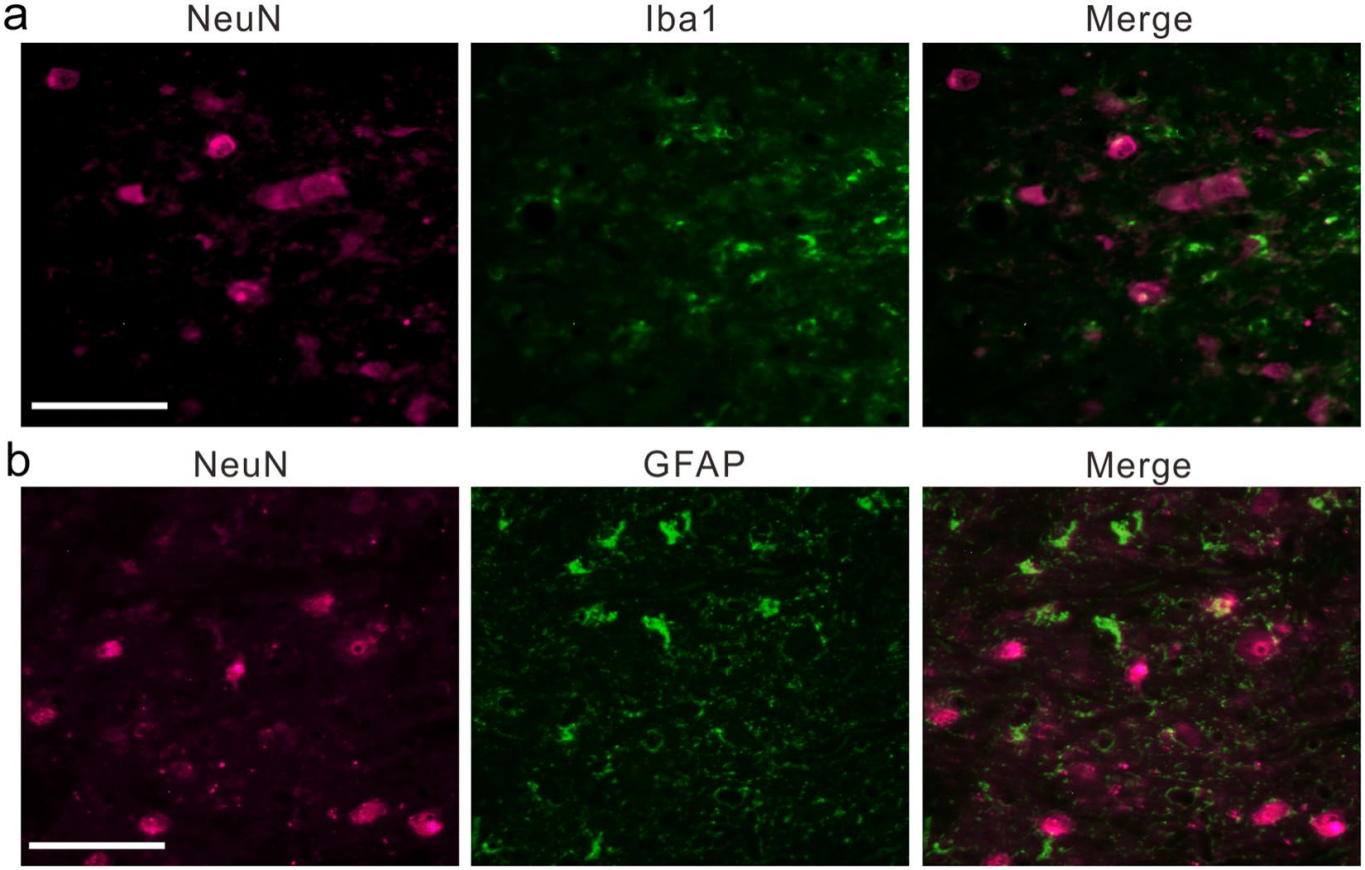
Surviving neurons in the perilesional area of the VPL were surrounded by processes of activated microglia and astrocytes. (**a**,**b**) Representative immunofluorescence images showing localizations of neuron (NeuN; magenta) and microglia (Iba1; green in **a**) and astrocyte (GFAP; green in **b**) at <200 μm from the border of the lesional area. Scale bar: 100 μm.

Finally, as a first step in investigating the histological changes induced by collagenase injection, we focused on glial activation because previous reports suggested that this activation may underlie abnormal pain after peripheral nerve injury^23, 26, 27, 30^. We performed immunohistochemistry for Iba-1 to investigate microglial proliferation and activation 3 months after collagenase injection and observed accumulation of Iba-1-positive cells around the histological lesions (Fig. 4a). Further, we observed cells with shapes characteristic of activated microglia^31, 32^, i.e., enlarged cells with thickened proximal processes and reduced ramification of distal branches (arrows in Fig. 4a). Iba-1-positive microglia were abundant not only in the lesional area, defined by a dense concentration of small cells (5–10 μm in diameter) in the Nissl-stained section, but also in the perilesional area, several hundred μm from the border of the lesional area (Fig. 4e). GFAP-positive astrocytes were also abundant in the perilesional area at 3 months after collagenase injection (Fig. 4c,f); we observed cells with shapes characteristic of activated astrocytes, i.e., enlarged cell bodies and thick processes^33^ (arrows in Fig. 4c). No activated microglia or astrocytes were detected in the VPL of the hemisphere contralateral to collagenase injection (Fig. 4b,d). We also performed immunohistochemistry for NeuN and confirmed the presence of neurons in the perilesional area, although the density of neurons was lower than that in the intact VPL (Fig. 4g). Double-labeling immunofluorescence indicated that both Iba-1-positive microglia and GFAP-positive astrocytes existed in the vicinity of neurons in the perilesional area, and the surviving neurons were surrounded by processes of the activated microglia and astrocytes (Fig. 5a,b).

## Discussion

In the present study, we developed a primate model of CPSP based on a hemorrhagic lesion induced in the VPL of rhesus macaques. The lesioned macaques showed behavioral changes that were interpreted as reflecting the development of both mechanical allodynia and thermal hyperalgesia.

In rodent models of CPSP, the onsets of mechanical allodynia and thermal hyperalgesia occur within 1 week after the induction of hemorrhage due to the injection of collagenase around the VPL^17, 18, 20^. In a majority of patients with CPSP, however, the onset of abnormal pain often occurs several weeks after a cerebrovascular accident^2^. A similar delay in the onset of CPSP was recently reported in rats in which a focal ischemic lesion in the thalamus was induced by injection of endothelin-1^34^. Unfortunately, this animal model exhibited thermal hyperalgesia but not mechanical allodynia, which is one of the most common symptoms of CPSP^35, 36^. The different behavioral changes in rodent CPSP models depending on the methods for lesion induction may reflect the different effects of hemorrhagic and ischemic strokes on the development of central pain. However, variations in lesion size might also contribute to the differences in behavioral changes, because lesions in the ischemic model were smaller than in the hemorrhagic models^17, 18, 20, 34^.

The present primate model, in which significant behavioral changes to both mechanical and thermal stimulation were observed several weeks after hemorrhage induction, reproduced the symptoms of CPSP more faithfully than did other experimental animal models using rodents. The difference between the present and previous models may reflect a species difference or differences in the size and location of the lesion. In the rodent models, the injection sites for collagenase type IV and endothelin-1 were determined using stereotaxic coordinates. In contrast, in the present study, the injection site was determined by the anatomical MR images of an individual macaque and electrophysiological recordings in which the location of the VPL sub-region responsive to tactile stimulation of the hand was identified. These techniques, in combination with the relatively large brain of rhesus macaques, contributed to inducing a hemorrhagic lesion more focal than those induced in the rodent models above. Note that the stroke area at 3 days after collagenase injection, when the stroke was largest, included several other thalamic nuclei, such as the dorsomedial and pulvinar nuclei. Therefore, plastic changes that result from damage to thalamic nuclei other than the VPL may continue even after the stroke region has stabilized and may participate to induce CPSP-like behavior. The present study is the first to use MRI to show the spatiotemporal changes of a stroke among animal models of CPSP and suggest that lesions based on histologic confirmation after the completion of behavioral assessments may be substantially underestimated because stroke size transiently increases and finally decreases after stroke induction.

In using this new primate model, an important next step will be to investigate how the location of the lesion affect the symptoms of CPSP. Anatomical brain imaging studies have been conducted to elucidate how lesion locations in patients with CPSP differ from those in patients with thalamic stroke without pain^4, 13, 37^. We believe that the present model, in which a small lesion can be made in the thalamic nucleus, will contribute to this understanding. For example, one drawback of the present macaque model is that the macaques did not display behavioral changes that were reflective of the development of cold hyperalgesia, a characteristic of CPSP; therefore, elucidating brain sites that are associated with this phenomenon is important.

Although we observed behavioral changes in the contra-lesional hand that were interpreted as signifying development of both mechanical allodynia and thermal hyperalgesia, behavioral changes interpreted as the development of mild thermal hyperalgesia were also observed in the ipsi-lesional hands. It has been reported that some patients with CPSP have sensory symptoms on the side ipsilateral to the lesion^38^. A recent study reported bilaterally spread cortical atrophy in patients with CPSP^39^, which may underlie the bilateral sensation of pain. The present model will aid in understanding changes in neuronal structures involved in the bilateral symptom.

Previous studies using rodent models have shown that microglial activation is associated with the pathophysiology of CPSP^17, 18^, and that pain-like behaviors are reduced by infusions of minocycline and P2 × 7 receptor antagonists^17, 21^, which are known to attenuate microglial activation^40, 41^. These results suggest that microglia are key players in the development of CPSP. The present study also showed microglial activation in the perilesional and lesional areas. Microglia were detected in the vicinity of neurons in the perilesional area and the surviving neurons were surrounded by microglial processes; therefore, microglia may enhance the excitability of neurons through extracellular signaling^42, 43^, which is suggested to be a mechanism involved in CPSP^21^. We also detected neurons in the perilesional area surrounded by processes of activated astrocytes, which exert an influence on the activity of surrounding neurons by interacting with activated microglia, as suggested by previous studies^25, 44^. The long-lasting activation of astrocytes, as well as microglia, suggests that microglia/astrocyte interaction in the perilesional area induces abnormal excitability of surviving neurons in the VPL, some of which convey noxious information^45^.

In contrast to the rodent models described above^17, 18^, in which significantly increased immunoreactivity for both microglial and astrocytic marker proteins in the perilesional area was confirmed for up to 1 month, the present results showed a significant increase that lasted for at least 3 months after collagenase injection. Together with the results of a previous study in rhesus macaques with a motor cortex lesion, in which microglial activation persisted for 12 months after the lesion^46^, the present results suggest that the temporal dynamics of glial activation and the resulting changes in brain activity and function differ between primates and rodents. Because reactive microglia were reported to be detectable in the injured human spinal cord even at one year after injury^47^, the present model using a non-human primate may be an important system to assess the role of prolonged gliosis occurring in human patients. We should point out that the present study did not demonstrate a causal relationship between microglial activation and the development of CPSP. An important next step in this regard is to ascertain whether the administration of minocycline, a microglial inhibitor, ameliorates CPSP, as occurs in the mouse model of CPSP^17^. The present model may have an advantage over other experimental models in testing therapeutic interventions that last for several months, such as continuous pharmacological treatment and rehabilitative training.

## Methods

### Subjects

Four male adult rhesus macaques (*Macaca mulatta*) were used in the present study (Table 1). We also used the brain tissues of three intact macaques weighing 5.1, 7.5 and 8.0 kg as the controls for immunohistochemical analysis (see below). The macaques were purchased from a local provider (Hamri Co., Ltd., Ibaraki, Japan). The animal use protocol was approved by the Institutional Animal Care and Use Committee of the National Institute of Advanced Industrial Science and Technology (AIST), and were carried out in accordance with guidelines within the “Guide for the Care and Use of Laboratory animals” (Eighth ed., National Academy of Science).

### Identification of VPL and collagenase injection

An artificial stroke was induced unilaterally in the VPL—in the right hemisphere for Macaques C and S, and the left for Macaques I and P (Table 1). After a scalp incision was made, the skull was exposed and two MR-compatible head posts were attached to the skull under sterile conditions and pentobarbital anesthesia (25 mg/kg), as in our previous study^48^. The location of the VPL was determined for each animal, using stereotaxic coordinates from MR images of each macaque’s brain obtained with a 3.0 T MRI system (Philips Ingenia 3.0 T, Philips Healthcare, Best, the Netherlands). Before the scan, the animals were anesthetized using medetomidine (0.05 mg/kg), midazolam (0.3 mg/kg), and ketamine (4 mg/kg), and then fixed in a magnet-free stereotaxic frame. The MRI protocols consisted of a T1-weighted turbo field echo sequence (repetition time (TR)/echo time (TE), 7.3/3.2 ms; number of excitations (NEX), 2; flip angle, 8°; field of view, 134 mm × 134 mm; matrix, 224 × 224; slice thickness, 0.6 mm; number of slices, 200) and a T2-weighted turbo spin echo sequence (TR/TE, 1500/283 ms; NEX, 2; flip angle, 90°; field of view, 134 mm × 134 mm; matrix, 224 × 224; slice thickness, 0.6 mm).

Following the MRI, the VPL sub-region involved in tactile sensation of the hand digits was identified by electrophysiological recordings, using procedures nearly identical to those used in our previous work^48, 49^. The microelectrode was penetrated at 1-mm intervals on the cortical surface, and the somatotopic organization of the VPL was determined by recording multiunit activity during light tactile stimuli applied to the contralateral body surface with a small hand-held brush. Collagenase type IV (C5138; Sigma, St. Louis, MO, USA; 200 U/ml in saline) was then injected in the identified VPL sub-region where neurons were activated by tactile stimuli. A single 4-μl injection of collagenase type IV was given to Macaque C, two 4-μl injections separated by 1 mm in the dorsoventral direction were given to Macaques I and P, and four 4-μl injections separated by 1 mm in both dorsoventral and rostrocaudal directions were given to Macaque S (Table 1).

### Behavioral procedures

We performed both mechanical and thermal withdrawal tests to evaluate pain perception on the hands after the collagenase injection into the VPL. During the test, the macaque sat in a primate chair made of acrylic glass, and the wrists of both forelimbs were fixed to a horizontal plane positioned at the height of the macaque’s waist. An opaque board was placed between the face and forelimbs; hence, the macaque did not see its own hand while mechanical or thermal stimuli were applied to it. A mechanical withdrawal test was conducted by using an electronic von Frey anesthesiometer (IITC Life Science, Inc., Woodland Hills, CA, USA). Each von Frey filament was applied perpendicularly to the palmar surface of the second, third, and fourth fingers, and the maximum pressure exerted (in grams) that triggered hand withdrawal was assessed. Two trials were performed for each finger, and the larger pressure was adopted as the datum for that day. The withdrawal thresholds for both hands, one contralateral and the other ipsilateral to the affected VPL (*i.e*., the contra-lesional and ipsi-lesional hands), were assessed 4 days/week during weeks 1 to 4 and 2 days/week during weeks 5 to 12 after collagenase injection, and then compared to those obtained before lesion induction. On the same day, a thermal withdrawal test was also conducted by using a thermal stimulator (SCP-85; As One Corporation, Osaka, Japan). The plate surface was maintained at 55–45 °C (for heat stimuli), 37 °C (for control stimuli) or 10–5 °C (for cold stimuli), and the macaque’s hand was transferred to the plate. Although the macaque freely escaped from hot stimuli, to avoid the risk of low-temperature burn injury from prolonged contact with the plate, the duration of a single trial was designed to be no more than 1 min. Multiple trials (maximum, five) were performed at each temperature until the total contact time with the plate exceeded 90 s. To evaluate the degree to which the thermal stimulus affected withdrawal latencies, the latency for thermal stimulation was divided by that for control stimulation at 37 °C. This ratio before lesion induction was compared to that after lesion induction for both hands. Hand withdrawal latencies were estimated in real time during the withdrawal test. In addition, the movements of both hands during the test were recorded, by using two digital video cameras (HC-V520M; Panasonic, Osaka, Japan) installed around the task apparatus, and a subset of the videos was re-analyzed by a person blinded to the treatment of the macaques. The results of this re-analysis were consistent with those of the real-time evaluations.

### Confirmation of lesion site

To evaluate the spatiotemporal changes of a stroke, anatomical MRI scans were performed before, and 1 day, 4 days, 1 week, 2 weeks, 1 month, and 3 months after collagenase injection. The MRI protocols with both T1- and T2-weighted sequences were the same as those described above. The areas that contained edema and hematoma were seen as a region of high and low signal intensities, i.e. hyperintense and hypointense areas, in T2-weighted MR images, respectively, as in previous studies^50–53^. The unbiased volumes for both edema and hematoma were calculated by Cavalieri’s principle^54^, using StereoInvestigator imaging software (MBF Bioscience, Williston, VT, USA). In the atlas-based analysis, the T2-weighted MR images of macaques that received a collagenase injection into the right hemisphere were flipped along the mid-sagittal plane, so that the stroke area was located on the left side of the brain. These processed MR images were matched to macaque brain template (voxel size, 0.5 × 0.5 × 0.5 mm)^55^ by using SPM12 software (http://www.fil.ion.ucl.ac.uk/spm/). After individual images were processed as described, the areas with hypointense signals were delineated manually as regions of interest and overlaid onto the template by using MRIcro software (http://www.mricro.com).

The size and location of the lesion induced by collagenase injection were also evaluated by histological analysis, using the procedure employed in our previous studies^48, 49^. After the Behavioral experiments were completed, frozen brain sections at 18 μm of thickness were prepared as in our previous studies^56–58^. Images of the Nissl-stained sections were photographed under an Olympus BX60 microscope by using a 3CCD color video camera (DV-47d; MBF Bioscience) and then digitized using StereoInvestigator imaging software. The lesioned area was defined as the area of a dense concentration of small cells (5–10 μm in diameter), which presumably incudes both glial cells and blood cells, and unbiased volumes were calculated using the same method as that for MR image analysis.

### Immunohistochemistry

To characterize the degree of microglial and astrocyte reactivity, as well as neuronal degeneration, 3 months after collagenase injection, we performed immunohistochemistry to detect ionized calcium binding adaptor molecule 1 (Iba-1), glial fibrillary acidic protein (GFAP), and neuronal nuclear antigen (NeuN) on brain sections obtained from Macaques C, I, S. Immunohistochemistry for each antigen was performed with a rabbit anti-Iba-1 polyclonal antibody (1:16,000; cat. no. 019–19741; Wako Pure Chemicals, Osaka, Japan), a rabbit anti-GFAP polyclonal antibody (1:125; cat. no. Z0334; Dako, Glostrup, Denmark) and a mouse anti-NeuN monoclonal antibody (1:50; cat. no. MAB377; Chemicon, Temecula, CA, USA). The immunocomplex was visualized by the avidin-biotin-peroxidase method. The Vectastain® Elite ABC Rabbit IgG Kit (PK-6101; Vector Laboratories, Inc., Burlingame, CA, USA) was used for Iba-1 and GFAP staining, whereas the Vectastain® Elite ABC Mouse IgG Kit (PK-6102; Vector Laboratories, Inc.) was used for NeuN staining. The color reaction was developed using diaminobenzidine (DAB) for the substrate (Dojindo Laboratories, Kumamoto, Japan) according to the manufacturer’s instructions. To investigate whether microglia and astrocytes were located in the vicinity of neurons in the perilesional VPL, double-labeling immunofluorescence was also performed as described previously^59^. In the double-labeling experiments, either the anti-Iba-1 or anti-GFAP antibody was incubated with the anti-NeuN antibody, and then visualized using Alexa 488-conjugated goat anti-rabbit IgG and Alexa 546-conjugated goat anti-mouse IgG (Invitrogen, Waltham, MA, USA). Sections were washed and then covered with Fluoromount/Plus™ (Diagnostic Biosystems, Pleasanton, CA, USA).

The immunoreactivity in the perilesional area was quantified using an analysis method modified from that used in a previous study^60^. The relative optical density (OD) for each antigen was measured in a 100 µm × 100 µm square that was randomly sampled around the border area of the lesion using Image J software (National Institutes of Health, Bethesda, MD, USA). The OD was also measured in 200 µm × 200 µm squares sampled at distances 100–300, 300–500, 500–700, 700–900, 900–1,100, and 1,100–1,300 µm from the border. For each antigen, 10 squares were measured for each distance, and the OD of the background staining was also measured in ten 200 µm × 200 µm squares that sampled the neighboring white matter in each section. We evaluated the staining intensity by calculating the percentage of OD of each square in the perilesional VPL above the background level. Then, the OD value in the perilesional VPL was normalized to the values obtained from the VPL of the contra-lesional hemisphere and those in the corresponding VPL of normal intact macaques.

### Statistical analysis

All data are presented as medians and interquartile ranges. Statistical significance was assessed by nonparametric tests including a two-tailed Mann–Whitney U-test, Wilcoxon signed-rank test, and Kruskal–Wallis one-way analysis of variance (ANOVA) with Dunn’s *post hoc* test. Statistical results were considered significant at *P* < 0.05. The statistical analyses were performed using GraphPad Prism software (GraphPad Software Inc., San Diego, CA, USA).

## Electronic supplementary material

Supplementary video S1

Supplementary video S2
